# LONELINESS AMONG PERSONS WITH LONGSTANDING MULTIPLE SCLEROSIS (MS)

**DOI:** 10.1093/geroni/igae098.2540

**Published:** 2024-12-31

**Authors:** Alexa Stuifbergen, Darla Grimes, Heather Becker, Nani Kim

**Affiliations:** The University of Texas Austin, Austin, Texas, United States; The University of Texas Austin, Austin, Texas, United States; The University of Texas Austin, Austin, Texas, United States; The University of Texas Austin, Austin, Texas, United States

## Abstract

The isolation induced by the global COVID pandemic highlighted the negative health and quality of life impact of loneliness and loss of social connections, especially among those who are aging with chronic disabling conditions with associated mobility and sensory impairments that make community participation more difficult. The purpose of this study was to explore the relationship between loneliness and depressive symptoms and health behaviors and quality of life outcomes for persons with MS using data collected in 2023 as part of an ongoing longitudinal survey study. The survey included measures of loneliness, depressive symptoms, self-rated health, health behaviors and quality of life outcomes. The sample (N=157) of community-residing persons with MS had a mean age of 70.70 (SD=7.80) and had been diagnosed with MS for an average of 35.16 years (SD=6.16). The majority were married (61%) and female (90%). Fifty-five percent of participants rated their overall health as good or excellent. Reports of loneliness were strongly positively correlated with reports of depressive symptoms (r=.63, p<.001), negatively correlated with economic adequacy (r= -.64, p<.001) and had a small negative correlation with age (-.23, p< 01). Greater functional limitations were associated with greater loneliness (r=.27, p<.001) and depressive symptoms (r=.37, p<.001). Loneliness and depressive symptoms had a similar pattern of moderate to strong negative correlations with reports of health behaviors and quality of life outcomes. Novel interventions to identify and treat loneliness and depression among older adults with chronic disabling conditions may improve important health and quality of life outcomes.

